# Battle Royale: Innate Recognition of Poxviruses and Viral Immune Evasion

**DOI:** 10.3390/biomedicines9070765

**Published:** 2021-07-01

**Authors:** Huibin Yu, Ryan C. Bruneau, Greg Brennan, Stefan Rothenburg

**Affiliations:** Department of Medical Microbiology and Immunology, School of Medicine, University of California, Davis, CA 95618, USA; glbyu@ucdavis.edu (H.Y.); rcbruneau@ucdavis.edu (R.C.B.); gbrennan@ucdavis.edu (G.B.)

**Keywords:** poxviruses, vaccinia virus, pattern recognition receptors, PKR, RNase L, ZBP1, cGAS–STING, Toll-like receptors, inflammasome

## Abstract

Host pattern recognition receptors (PRRs) sense pathogen-associated molecular patterns (PAMPs), which are molecular signatures shared by different pathogens. Recognition of PAMPs by PRRs initiate innate immune responses via diverse signaling pathways. Over recent decades, advances in our knowledge of innate immune sensing have enhanced our understanding of the host immune response to poxviruses. Multiple PRR families have been implicated in poxvirus detection, mediating the initiation of signaling cascades, activation of transcription factors, and, ultimately, the expression of antiviral effectors. To counteract the host immune defense, poxviruses have evolved a variety of immunomodulators that have diverse strategies to disrupt or circumvent host antiviral responses triggered by PRRs. These interactions influence the outcomes of poxvirus infections. This review focuses on our current knowledge of the roles of PRRs in the recognition of poxviruses, their elicited antiviral effector functions, and how poxviral immunomodulators antagonize PRR-mediated host immune responses.

## 1. Introduction

Members of the family *Poxviridae* can infect a diverse range of vertebrates and invertebrates, although some poxviruses have narrow host ranges and others have very broad host ranges [1]. Poxvirus infections have posed serious threats to both humans and animals worldwide [2]. *Poxviridae* is a large family of DNA viruses comprised of two subfamilies: *Chordopoxvirinae* and *Entomopoxvirinae*. There are currently 18 recognized genera of *Chordopoxvirinae*, which infect vertebrates, and 4 genera of *Entomopoxvirinae*, which infect invertebrates [1]. Poxviruses possess a single, linear double-stranded DNA (dsDNA) genome, which ranges in size from 127 to 456 kb and encodes several hundred gene products. Unlike most other DNA viruses, poxviruses replicate exclusively within the cytoplasm of permissive cells [3].

One of the best known poxviruses is variola virus (VARV), a member of the orthopoxvirus genus. VARV is the causative agent of human smallpox, which was one of the most devastating human diseases in history [4]. Despite the success of global smallpox eradication through a vaccination campaign led by the World Health Organization, other pathogenic poxviruses, such as monkeypox virus, cowpox viruses (CPXV), camelpox virus, tanapox virus, and capripoxviruses remain threats to human and animal health [1,2,5,6]. The most intensively studied poxviruses, such as vaccinia virus (VACV) and myxoma virus (MYXV), have proven to be excellent research models to study host innate recognition and virus–host protein interactions and have provided important insights into the fields of virology and immunology [7,8]. In turn, these fundamental insights have direct translational applications to improve the development of safer and more effective attenuated viral vectors for vaccines, cancer therapeutics, and other treatment modalities [9]. For example, in VACV inactivation of either IL-1β-binding protein or IL-18-binding protein, encoded by B15R and C12L, respectively, enhanced CD8^+^ T cell memory responses after immunization and improved the protection against virulent VACV WR challenge [10,11]. In addition, VACV has the genomic capacity to incorporate >25 kb of foreign DNA without noticeable impacts on viral replication. This capacity has been employed to genetically engineer VACV chimeras carrying multiple heterologous genes, as both polyvalent vaccines and treatment of various genetic diseases [12,13,14,15]. Multiple poxviruses are being investigated for use in oncolytic virotherapy. Myxoma virus (MYXV) is one such preclinical candidate oncolytic virus, and recombinant MYXV lacking various viral death modulator genes, such as M-T5 [16], M11 [17], M13 [18], and Serp2 [19], have enhanced anti-tumor activity that appears to be mediated through viral induction of programmed cell death rather than through viral replication (reviewed in [20]).

The recognition of viral pathogens and the host defense against them are provided by the innate and adaptive immune systems. The adaptive immune system is broadly comprised of antigen-specific CD8^+^ T cells, CD4^+^ helper T cells, and B cell antibody responses for specific, anamnestic protection against distinct pathogens [21,22,23]. Prior to the initiation of the adaptive immune response, pathogen-associated molecular patterns (PAMPs) derived from poxviruses, such as DNA and RNA, as well as envelope or core proteins, can be sensed by a diverse set of pattern recognition receptors (PRRs) to initiate the faster but less specific innate immune responses [24,25,26,27,28,29,30,31]. The innate immune response provides the first line of host defense and includes antiviral proteins that can lead to the direct elimination of viruses or induce the expression of type I interferons (IFNs), proinflammatory cytokines, chemokines, and other antiviral proteins [32,33]. These effector molecules mediate direct antiviral effects or orchestrate the adaptive immune response to contain poxvirus infections at various stages. In particular, type I IFNs, the hallmark effector of antiviral responses, are essential to initiate innate immunity and also to mediate the subsequent development of adaptive immunity against invading poxviruses [34]. In addition, type I IFNs upregulate the expression of hundreds of IFN-stimulated genes (ISGs) that directly influence protein synthesis, cell growth, and survival to establish an antiviral state [35,36]. Furthermore, multiple cytokines, such as interleukin-6 (IL-6), IL-12, and tumor necrosis factor-alpha (TNFα), can be induced during poxvirus infections which then act systemically to induce immune responses [37,38].

Over recent decades, substantial progress has been made in defining the roles of PRRs and the subsequent signaling pathways that are involved in the sensing of and response to poxviruses. IFN expression is transcriptionally regulated through activation of IFN regulatory factor (IRF) family members or coordinated activation of IRFs and nuclear factor kappa B (NF-κB) [39]. The stimulation of proinflammatory genes depends on activation of the transcription factors NF-κB and activator protein 1 (AP1) [40]. Despite the diversity of PRR ligands, many PRR-regulated signaling pathways share common downstream molecules, such as myeloid differentiation primary response gene 88 (MyD88) and Toll/interleukin-1 receptor domain-containing adapter-inducing interferon-β (TRIF). Thus, there is substantial crosstalk and overlap between the signaling cascades stimulated by different PRRs, which lead to IFN activation and also drive the production of other cytokines [41,42,43].

A broad spectrum of PRRs has been implicated in poxvirus recognition, including RNA sensors, cytosolic DNA sensors, multiple Toll-like receptors (TLRs), and components of the inflammasome. In order to establish successful infections in the face of this multi-pronged immune response, poxviruses evade host antiviral responses by expressing a variety of viral proteins. Those viral proteins interact with and antagonize the key components of these intracellular signal transduction pathways. In this review, we discuss our current knowledge concerning how innate receptors or sensors detect poxviruses and how PRR-mediated recognition translates into effective antiviral immune responses. Following each sensor, we present mechanisms employed by poxviruses to thwart PAMP recognition by the host and how poxviruses manipulate PRR-mediated signaling pathways to their benefit.

## 2. Double-Stranded RNA-Activated Sensors and Poxvirus Antagonists

**Figure 1:**
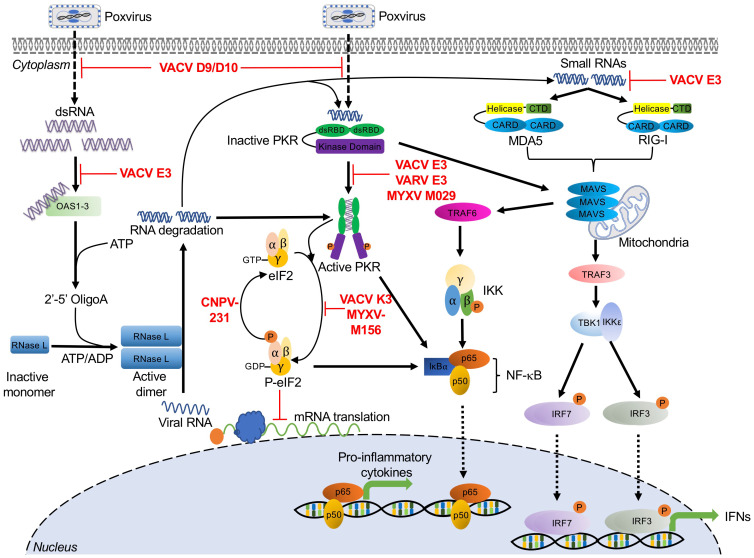
dsRNA sensor-mediated signaling pathways and poxvirus antagonists. The figure presents host sensors (black text) involved in recognizing dsRNA species from poxviral infections, and the elicited signaling cascades by these sensors, which are indicated by black arrows. Poxvirus-encoded immunomodulatory proteins that inhibit activation of these host pathways are indicated in red text and their effects on pathways are indicated by red lines. See main text for corresponding details and the underlying molecular mechanisms. Abbreviations used in this figure include ADP: adenosine diphosphate; ATP: adenosine triphosphate; CARD: caspase activation and recruitment domains; CNPV: canarypox virus; CTD: carboxy-terminal domain; dsRBD: dsRNA binding domain; dsRNA: double-stranded RNA; eIF2: eukaryotic translation initiation factor 2; GDP: guanosine diphosphate; GTP: guanosine-5′-triphosphate; IKKα: IκBα kinase α; IKKβ: IκBα kinase β; IKKε: IκBα kinase ε; IKKγ: IκBα kinase γ; IL-6: interleukin-6; IRF3/7: interferon regulatory factor 3/7; IκBα: inhibitor κBα; MAVS: mitochondrial antiviral-signaling protein; MDA5: melanoma differentiation-associated protein 5; MYXV: myxoma virus; NF-κB: nuclear factor kappa B; OAS: 2′-5′-oligoadenylate synthetases; p65/p50: NF-κB heterodimer p50/p65 subunit; PKR: protein kinase R; RIG-I: retinoic acid-inducible gene I; RNase L: ribonuclease L; TBK1: TRAF family member-associated NF-κB activator (TANK)-binding kinase 1; TNFα: tumor necrosis factor-alpha; TRAF3/6: tumor necrosis factor receptor-associated factor 3/6; VACV: vaccinia virus; VARV: variola virus.

The lifecycle of most virus families generates double-stranded RNA (dsRNA). In *Poxviridae*, dsRNA is the result of overlapping transcripts, which form duplexes, primarily at intermediate and late time points in the viral replication cycle [44,45,46]. Due to the ubiquity of this PAMP, it is perhaps not surprising that the first PRRs to be described were the dsRNA-activated molecules PKR and OAS/RNase L [35,47,48,49,50,51]. Since these initial discoveries, other broadly acting antiviral dsRNA sensors have been identified, and we have developed a better understanding of the plethora of mechanisms that viruses employ to evade these host proteins (Figure 1). In this section, we describe the mechanisms that these host proteins use to sense dsRNA, the antiviral pathways that they initiate, and the strategies poxviruses employ to inhibit them.

### 2.1. Protein Kinase R

Protein kinase R (PKR) was first discovered as an IFN-stimulated, dsRNA-dependent protein kinase [35,47,48]. PKR is comprised of two N-terminal dsRNA binding domains (dsRBDs) and a C-terminal catalytic kinase domain [52]. These functional domains allow PKR to serve as both an intracellular sensor for double-stranded RNA (dsRNA) and an effector serine/threonine protein kinase. PKR is expressed in most cell types as an inactive monomer at intermediate levels. PKR recognizes dsRNA via its dsRBDs, and this binding event leads to PKR dimerization and autophosphorylation [53,54]. Activated PKR phosphorylates the alpha subunit of the eukaryotic translation initiation factor 2 (eIF2), which forms the ternary complex together with initiator methionyl-tRNA and GTP. Concomitant with translation initiation, GTP is hydrolyzed to GDP, which must subsequently be exchanged with GTP by the guanine nucleotide exchange factor eIF2B (reviewed in [55]). However, phosphorylated eIF2α has increased binding affinity between eIF2 and eIF2B. This increased affinity effectively turns eIF2α into an inhibitor of eIF2B, thus impairing the generation of active GTP-bound eIF2 and inhibiting the initiation of cap-dependent translation, ultimately leading to cell death and inhibition of viral replication (Figure 1) [56,57,58,59].

In addition to its canonical function as an eIF2α kinase, PKR has also been implicated in different stress-induced signaling pathways including IFN responses and NF-κB-dependent inflammatory responses [60,61,62]. It has been reported that PKR activation decreases the protein levels of IκBα, the NF-κB inhibitor, thus activating this pathway. The molecular mechanism underlying this activation appears to rely on the kinase activity of PKR which has been shown to be essential for NF-κB activation [61,63]. Additionally, during infection with VVΔE3L, a VACV lacking the PKR antagonist protein E3, PKR was shown to regulate melanoma differentiation-associated protein 5 (MDA5)-mediated IFNβ production through a mechanism that does not require eIF2α phosphorylation. Interestingly, PKR activation also upregulated IFN induction by MAVS (mitochondrial antiviral-signaling protein) in an MDA5-independent manner [64]. Similarly, in human and murine cells infected with a modified vaccinia virus Ankara (MVA) strain engineered to produce more dsRNA, the expression of IFNβ increased. Furthermore, mice infected with this MVA strain showed increased expression of IFNα, IFNγ, and other cytokines, which were enhanced in a PKR-dependent manner [27].

#### Poxvirus Evasion of PKR

Poxviruses encode diverse PKR antagonists that either directly or indirectly inhibit the PKR pathway. In VACV, these proteins are encoded by *E3L*, *K3L*, *D9R*, and *D10R*. In addition, avipoxviruses possess homologs of cellular protein phosphatase 1 (PP1) targeting growth arrest and DNA damage-inducible protein 34 (GADD34, PPP1R15A) and constitutive repressor of eIF2α phosphorylation (CREP, PPP1R15B), which promote the dephosphorylation of eIF2α (Figure 1) [65].

E3 was initially characterized as a dsRNA-binding protein responsible for IFN resistance. This phenotype was dependent on the C-terminal domain of E3, which contains a dsRBD [66]. E3 can bind dsRNA via this dsRBD to prevent PKR dimerization and activation [67,68]. In human-derived HeLa cells, an E3L-deleted VACV displayed a replication defect [69]. Moreover, this E3L-deleted VACV was able to replicate in PKR-deficient HeLa cells as efficiently as wild type VACV, which demonstrated that PKR was the major target of E3 in these cells [70]. However, VACVΔE3L replication in Syrian hamster-derived BHK cells was not affected, suggesting that, as with most other host range genes, there is a species-specific component to E3 activity. Additionally, E3 orthologs from myxoma virus and swinepox virus were shown to inhibit PKR activity and impair the induction of IFNβ and proinflammatory cytokines, such as TNFα and IL-6, during viral infection [68,71]. Interestingly, E3L orthologs from myxoma virus and swinepox, but not from sheeppox virus, were able to rescue virus replication when expressed by a VACVΔE3L chimera. These PKR-inhibitory activities correlated with PKR inhibition in vitro studies as well, further supporting the hypothesis that there are ortholog-specific differences in E3 activity [68,72].

VACV K3 and its orthologs in other poxviruses are structural mimics of eIF2α. They act as pseudosubstrate inhibitors and compete with eIF2α to bind PKR, thus preventing eIF2α phosphorylation and allowing protein synthesis to continue [73,74,75]. As with E3L, K3L was also identified as a VACV host range gene when a K3L-deleted VACV was able to replicate in HeLa cells but not in BHK cells [69]. Further evidence for this host range function is provided by the observation that K3 orthologs from multiple poxvirus genera exhibited both virus- and host-specific inhibition of PKR in both reporter- and infection-based assays [72,76,77,78,79].

VACV encodes two decapping enzymes D9 and D10, which share approximately 25% amino acid sequence identity [80,81]. D9 and D10 are expressed either early or late in infection, respectively. They cleave m^7^GDP from capped RNA substrates through nudix hydrolase motifs. This decapping activity reduced mRNA stability and prevented dsRNA accumulation, thereby indirectly preventing PKR activation. Inactivation of D9 and D10 catalytic activities increased PKR-induced eIF2α phosphorylation and enhanced PKR- and OAS/RNase L-mediated antiviral responses during VACV infection [81,82,83,84].

E3 and K3 orthologs evolved in mammalian poxviruses after the split from avipoxviruses [85]. In order to inhibit PKR, avipoxviruses evolved inhibitors that act later in the PKR pathway, facilitating the dephosphorylation of eIF2α. These proteins are homologous to the PP1 adaptor proteins GADD34 and CREP. In a yeast-based system, the canarypox virus protein 231 reversed the cytotoxic effects of ectopically expressed human PKR and reduced the level of PKR-induced eIF2α phosphorylation [65]. The roles of avipoxviral GADD34 homologs in poxvirus infection still need to be elucidated.

### 2.2. 2′-5′-Oligoadenylate Synthetase (OAS)/RNase L

The 2′-5′-oligoadenylate synthetase (OAS)/RNase L-mediated antiviral pathway was discovered as one of the first interferon-induced systems in response to diverse viral infections [36]. The OAS family includes OAS1, OAS2, and OAS3 and their expression can be upregulated by type I and type III IFNs [86]. These molecules detect accumulated dsRNA species from diverse sources, which are usually viral but may sometimes have cellular origins [87,88]. Upon binding dsRNA, enzymatically active OAS1, OAS2, and OAS3 use ATP to synthesize linear 2′-5′-linked second messenger molecules called 2′-5′-oligoadenylates (2-5As) [36,89]. 2-5As, in turn, bind and activate latent RNase L monomers in the cytoplasm, inducing RNase L dimerization and activation. Activated RNase L suppresses viral replication by cleaving viral and cellular RNAs, limiting mRNA translation and promoting apoptosis (Figure 1). The resulting RNA cleavage products can be recognized by other RNA sensors, such as PKR, retinoic acid-inducible gene I (RIG-I), and MDA5 (Figure 1). Thus, RNase L acts to both inhibit viral replication itself and amplify the response of other innate immune proteins [90,91,92,93]. For example, RNase L activation has been shown to amplify IRF3-dependent IFN production by inducing the formation of antiviral stress granules, for which PKR and RIG-I are essential mediators [93].

Multiple studies have demonstrated that the antiviral activity of OAS/RNase L limits poxvirus infections. In response to VACV infection, RNase L knockout (KO) C57BL/6 mice were more susceptible to viral infection than wild type mice, indicating that RNase L plays an antiviral role in vivo [28]. Recombinant VACV expressing OAS or RNase L showed impaired viral replication relative to wild type VACV. This replication defect was accompanied by increased rRNA degradation and inhibition of virus protein synthesis [94]. In A549 cells, OAS3, but not OAS1 or OAS2, played the dominant role in RNase L activation and subsequent antiviral effects in response to VACV infection [89].

#### Poxvirus Evasion of OAS/RNase L

VACV E3 has been shown to inhibit the activation of the OAS/RNase L system [95]. Compared to MVA infection, HeLa cells infected with MVA-∆E3L inhibited viral replication at multiple steps including viral late transcription, late mRNA translation, and viral DNA replication. These replication blocks were associated with activation of OAS/RNase L, rRNA degradation, and upregulation of host transcripts, such as IL-6 [96]. Furthermore, VACVΔE3L replicated approximately 20-fold higher in RNase L^KO^ MEF cells than in cells with intact RNase L, suggesting that E3 is involved in suppression of the OAS/RNase L system [97]. As described above, the VACV decapping enzymes D9 and D10 reduce dsRNA accumulation and thus also inhibit OAS/RNase L-mediated antiviral responses [81,83,98]. In A549 cells, knocking out both RNase L and PKR was necessary to allow replication of VACVΔE3L, whereas in HAP1 cells, PKR knockout alone was sufficient to allow VACVΔE3L replication. These data highlight the cell type-specific activities of the OAS/RNase L pathway [99].

### 2.3. RIG-I and MDA5

Retinoic acid-inducible gene I (RIG-I) and melanoma differentiation-associated protein 5 (MDA5) are intracellular pathogen sensors and type I IFN inducers, which belong to the RIG-I-like receptors (RLRs) family [100,101]. RIG-I and MDA5 are localized in the cytosol of most cell types, where they recognize dsRNA derived from a variety of viral infections [64,102,103,104,105,106,107,108]. Both RIG-I and MDA5 contain a carboxy-terminal domain (CTD), two central helicase domains (Hel1 and Hel2), and two N-terminal caspase activation and recruitment domains (CARDs) [109]. Despite these similarities, RIG-I and MDA5 show different RNA binding preferences. RIG-I primarily senses and recognizes short RNA ligands containing 5′ triphosphate groups, while MDA5 mainly recognizes long dsRNA and replication intermediates [110,111,112,113]. Once activated, RIG-I and MDA5 both initiate signaling cascades through the adaptor protein MAVS. MAVS forms a multilayered complex to mediate downstream signal transduction, inducing type I interferons (IFNs) through IRF3 and IRF7 phosphorylation, and activating NF-κB through the tumor necrosis factor receptor (TNFR)-associated factor 6 (TRAF6)-mediated signal cascade (Figure 1) [112,114,115].

During poxvirus infections, RIG-I has been shown to play an essential role in sensing MYXV and triggering the induction of TNFα and type I IFN in primary human macrophages in an IRF3- and IRF7-dependent manner [107]. Furthermore, in the presence of VACV DNA, RNA polymerase III exerts its antiviral effect by generating 5′ppp RNA, which acts as a RIG-I substrate [106]. Additionally, VACV late RNA transcripts can be sensed by either RIG-I or MDA5 in a cell type-specific manner, triggering IFNβ gene transcription [116]. However, during MVA infection, MDA-5, but not RIG-I, was essential for the induction of IFNβ mRNA responses in THP-1 cells [29]. Although this activation specifically by late transcripts is intriguing, it remains unclear which poxviral RNA structures or motifs activate RIG-I and/or MDA5.

#### Poxvirus Evasion of RIG-I and MDA5

When infected with VACVΔE3L, mouse primary keratinocytes produced IFNβ, IL-6, and other cytokines in a MAVS- and IRF-3-dependent manner. MAVS and IRF3 are essential components of the RIG-I and MDA5 pathway. Production of these cytokines was completely prevented by infection with wild type VACV, or by VACV expressing only the E3 dsRNA binding domain [108]. Similarly, a second mechanism of E3 inhibition of RIG-I has been proposed that is mediated by inhibiting RIG-I recognition of RNA generated by RNA polymerase III [117].

## 3. Dual RNA/DNA Sensor

### Z-DNA Binding Protein 1

**Figure 2:**
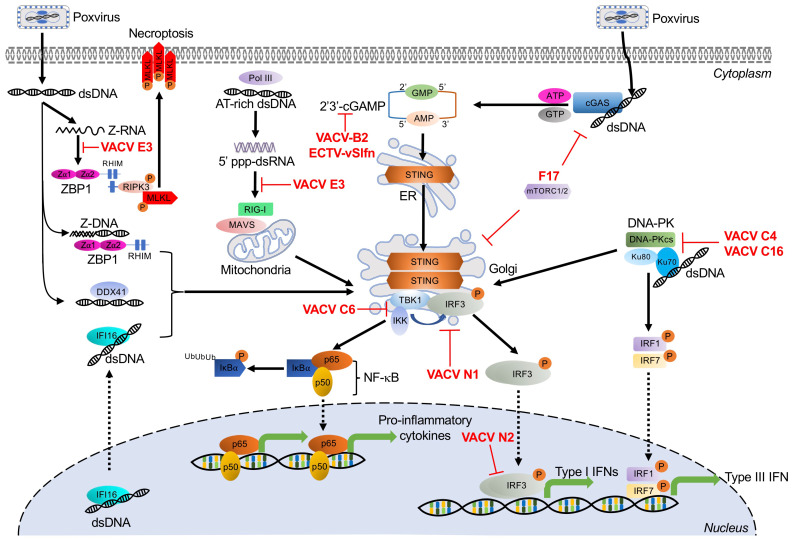
Cytosolic DNA sensor-mediated signaling pathways and poxvirus antagonists. DNA sensors are denoted in black text and the transduction of their triggered signaling cascades are indicated by black arrows. The diverse poxviral inhibitors of these cytosolic DNA sensors are indicated in red. See main text for corresponding details and the underlying molecular mechanisms. Abbreviations used in this figure include ADP: adenosine diphosphate; AMP: adenosine monophosphate; ATP: adenosine triphosphate; cGAS: cyclic GMP-AMP synthase; DDX41: Asp-Glu-Ala-Asp (DEAD) box polypeptide 41; DNA-PK: DNA-dependent protein kinase; DNA-PKcs: DNA-dependent protein kinase catalytic subunit; dsDNA: double-stranded DNA; ECTV: ectromelia virus; ER: endoplasmic reticulum; GMP: guanosine monophosphate; IFI16: interferon-γ inducible protein 16; IL-6: interleukin-6; IRF1/3/7: interferon regulatory factor 1/3/7; IκBα: inhibitor κBα; MAVS: mitochondrial antiviral-signaling protein; MLKL: mixed lineage kinase-like; mTORC1/2: mammalian target of rapamycin complex 1/2; MVA: modified vaccinia virus Ankara; NF-κB: nuclear factor kappa B; p50/p65: NF-κB heterodimer p50/p65 subunit; RHIM: RIP homotypic interaction motif; RIG-I: retinoic acid-inducible gene I; RIPK: receptor interacting protein kinase; RNA pol III: DNA-dependent RNA polymerase III; STING: stimulator of interferon genes; TBK1: TRAF family member-associated NF-κB activator (TANK)-binding kinase 1; TNFα: tumor necrosis factor-alpha; TRAF: tumor necrosis factor receptor-associated factor; VACV: vaccinia virus; vSlfn: viral Schlafen; ZBP1: Z-nucleic acid-binding protein 1; Zα: Z-nucleic acid binding domain; 2′3′ cGAMP: 2′3′ cyclic guanosine monophosphate–adenosine monophosphate; 5′ppp-dsRNA: 5′ triphosphate double-stranded RNA.

Z-DNA binding protein 1 (ZBP1), previously known as DLM-1 or DNA-dependent activator of IFN-regulatory factors (DAI), is a stress granule-associated protein, which contains two functional Z-DNA/RNA binding (Zα) domains in its N-terminus [118,119,120,121]. In addition to binding Z-DNA, it can also bind left-handed double-stranded Z-RNA and RNA, which adopt Z-RNA-like conformations [122]. ZBP1 has been identified as a putative cytosolic DNA sensor and activator for the induction of type I IFNs and other genes involved in innate immunity [123]. Upon binding DNA, ZBP1 dimerizes and recruits TANK-binding kinase 1 (TBK1) and IFN regulatory factor 3 (IRF3) to induce the production of type I IFNs and the NF-κB pathway (Figure 2) [123,124,125]. Activated ZBP1 can also activate receptor interacting protein kinase 3 (RIPK3) by binding through RIP homotypic interaction motifs (RHIM). This interaction leads to the activation of the mixed lineage kinase-like protein (MLKL), ultimately inducing necroptotic cell death (Figure 2) [126,127,128,129,130]. However, it is still unclear what specific ligand generated during VACV infection is recognized by ZBP1.

#### Poxvirus Evasion of ZBP1

VACV E3 contains an amino-terminal Zα domain that is homologous to the ZBP1 Zα domains. E3 is critical for VACV pathogenicity in vivo and for inhibition of the IFN response [97,131,132]. It has been proposed that E3 competes with ZBP1 for Z-nucleic acid binding in VACV-infected cells. This hypothesis is supported by the observation that E3 overexpression reduced DNA-mediated induction of IFNβ responses [124]. In addition, a VACV E3 mutant that lacks the ability to bind Z-DNA is less pathogenic in a wild type mouse infection model, but not in either ZBP1- or RIPK3-deficient mice. However, this pathogenicity could be restored by an E3 chimera expressing the first Zα domain of ZBP1 or ADAR1 [126,132]. Overexpression of E3 was also described to reduce DNA-mediated induction of IFNβ responses [124]. During VACV infection, only full-length E3, but not a Zα-deleted E3 mutant (Δ83N), prevented ZBP1-mediated RIPK3-dependent necroptosis. Importantly, VACV-E3LΔ83N showed strong attenuation in wild type mice, but not in either ZBP1-, RIPK3-, or DAI-deficient mice [126]. While most mammalian poxviruses contain E3L orthologs, some of these orthologs, including those from monkeypox virus and myxoma virus, do not encode functional Zα domains [85]. In myxoma virus and rabbit fibroma virus, which only productively infect rabbits and hares, the complete Zα domain-encoding DNA is missing from their E3 orthologs [85] and is therefore not predicted to inhibit RIPK3-dependent necroptosis.

## 4. DNA-Activated Sensors and Poxvirus Antagonists

Cytosolic DNA sensing mainly induces transcription of type I interferons but can also initiate NF-κB-dependent proinflammatory cytokines, which constitute an important frontline of antiviral defense against DNA viruses. Initial research demonstrated that multiple ligands, such as long poly(dA:dT), and dsDNA oligonucleotides led to the activation of the IRF3 pathway and subsequent type I interferon responses [133,134]. More recently, investigations have focused on defining the upstream DNA receptors, and this effort has advanced considerably over the past decade [135]. Along with TLR9, discussed in a separate section, this effort has led to the identification of multiple DNA receptors, of which cyclic GMP-AMP synthase (cGAS), DNA-dependent RNA polymerase III (Pol-III), interferon-γ inducible protein 16 (IFI16), DNA-dependent protein kinase (DNA-PK), and DEAD box polypeptide 41 (DDX41) have been implicated in detecting poxviruses (Figure 2).

### 4.1. Cyclic GMP-AMP Synthase

Cyclic GMP-AMP synthase (cGAS) belongs to the oligoadenylate synthase (OAS) protein family and recognizes DNA in the cytosol [136]. Upon binding DNA, cGAS dimerizes and catalyzes the synthesis of 2′,3′ cyclic guanosine monophosphate–adenosine monophosphate (2′, 3′ cGAMP), a 2′-5′-linked cyclic dinucleotide second messenger [137]. cGAMP binds to and activates the adaptor protein STING (stimulator of interferon genes). In turn, STING activates the protein kinases IκBα kinase (IKK) and TANK-binding kinase 1 (TBK1), leading to the induction of interferons and cytokines through activation of NF-κB and IRF3, respectively (Figure 2) [136,138,139,140,141].

The cGAS–STING axis is an important recognition pathway for multiple DNA viruses, including poxviruses. In ectromelia virus (ECTV)-infected inflammatory monocytes, STING played an essential role in inducing interferon production through the activation of IRF7 and NF-κB signaling. Mice deficient in IRF7- and NF-κB were susceptible to ECTV infection [142]. Additional work demonstrated that cGAS is a key sensor for ECTV infection. Knockdown of either cGAS or STING decreased transcription of IFNα and IFNβ in L929 cells infected with ECTV. This phenotype was also observed in cGAS- or STING-deficient mice [143]. The cGAS–STING axis has also been shown to detect VACV infection. IFNβ induction after VACV infection was largely abolished in cGAS- or STING-deficient mouse lung fibroblasts and dendritic cells [144]. In line with this observation, the cGAS–STING axis was necessary for type I IFN production in MVA-infected murine bone marrow-derived cDCs. Furthermore, cGAS and STING deficiency abolished TBK1 and IRF3 phosphorylation in these cells, and mice lacking STING or IRF3 showed decreased type I IFN expression compared to wild type mice in response to MVA infection [145].

#### 4.1.1. Poxvirus Evasion of cGAS

Poxviruses encode several proteins that target cGAS–STING at different steps in the pathway (Figure 2). STING phosphorylation and dimerization were suppressed during VACV-COP and -WR infection, indicating the existence of inhibitors that target this pathway upstream of STING [146]. The poxvirus protein F17, a late structural protein, has been reported to help evade cytosolic cGAS sensing. In response to infection with VACV lacking F17, cGAS mediated IRF activation and interferon-stimulated gene (ISG) responses in both macrophages and lung fibroblasts [147]. F17 bound and sequestered Raptor and Rictor, which are regulators of mammalian target of rapamycin complexes mTORC1 and mTORC2, respectively. F17-mediated mTOR dysregulation blocked STING-mediated ISG induction and antiviral responses, in part, through mTOR-dependent cGAS degradation [148]. More recently, VACV B2 was shown to degrade the second messenger 2′,3′-cGAMP, and this family of enzymes has been named “poxins”. Deleting VACV poxin led to significant attenuation in a skin infection mouse model, although, interestingly, IFNβ levels were not increased, suggesting that poxins act by preventing cGAMP spread more than they prevent downstream effector production [149].

Poxin-encoding genes are found in many other poxviruses but are notably inactivated in VARV and VACV-MVA. In VACV and the closely related horsepox and rabbitpox viruses, the poxin domain is found by itself. However, in most orthopoxviruses, the poxin domain is found in combination with a Schlafen (Slfn) family-related domain [149]. In ECTV, this protein was called vSlfn. vSlfn-deficient ECTV was strongly attenuated in mouse infection models and unable to block the activation of STING, TBK1, and IRF3 in macrophages and correlated with a strong IFN response [150].

#### 4.1.2. Poxvirus Evasion of STING

It was found that DNA-induced activation of STING and IRF3 can be inhibited by infection with cowpox virus, ECTV, and VACV strains Copenhagen (COP) and Western Reserve (WR), but not by MVA [146]. Both TBK1 and IRF3 are key downstream components of the STING pathway, which mediate the IFN response. These downstream effectors are also targeted by poxviral antagonists such as VACV-C6, N1, N2, and E3 proteins (Figure 2). VACV-C6, N1, and N2 belong to a family of B cell CLL/lymphoma 2 (Bcl-2)-like proteins. C6 inhibits the IFNβ response induced by poly(dA:dT) DNA by blocking the activation of TBK1 and IKKε while inhibiting translocation and activation of IRF3 [151]. VACV N1 was shown to directly associate with TBK1 to inhibit IRF3-mediated IFNβ responses. As an example of this effect, repairing the defective N1L gene in MVA abrogated the type I IFN response relative to infection with unaltered MVA. This IFN reduction coincided with reduced levels of TBK1 and IRF3 phosphorylation [145]. The early nuclear protein N2 acts further downstream in this pathway, inhibiting an IRF3-specific reporter (ISG56) response that was induced by overexpression of TBK1 or by poly(dA:dT) DNA stimulation. In addition, N2 also inhibited TBK1-elicited IFNβ promoter responses [152].

### 4.2. RNA Polymerase III

DNA-dependent RNA polymerase III (Pol III) is a ubiquitous enzyme, which mainly resides in the nucleus, where it fulfills most of its cellular functions, including transcription of short untranslated RNAs, including transfer RNAs, 5S rRNA, and U6 spliceosomal RNA, using DNA as a template. However, Pol III may also localize to the cytosol, where it acts as a DNA sensor and engages the RIG-I-mediated pathway [106]. In the cytosol, Pol III serves as a DNA sensor of several pathogens, including Legionella pneumophila, herpes simplex virus 1 (HSV-1), Epstein–Barr virus, and VACV [106,117]. It detects cytosolic AT-rich dsDNA through its DNA binding regions. Pol III then transcribes the DNA template into 5′-triphosphate-containing dsRNA, which activates RIG-I and leads to the induction of type I interferons and NF-κB activation (Figure 2) [106,153,154].

#### Poxvirus Evasion of Pol III

To date, the only described inhibitor of Pol III dsDNA sensing is VACV E3. In 293T cells transfected with full-length E3, poly(dA:dT) DNA induced IFNβ expression, and NF-κB activity was abolished. Furthermore, the dsRBD-containing C-terminus alone was sufficient to inhibit these responses, whereas the Zα domain-containing N-terminus was dispensable [117].

### 4.3. Interferon-γ Inducible Protein 16

The intracellular DNA sensor interferon-γ inducible protein 16 (IFI16) belongs to the pyrin and HIN200 domain (PYHIN) protein family. Although IFI16 contains a nuclear localization signal and is mainly located in the nucleus, it can also be found in the cytosol, where it can recognize single-stranded (ss) DNA and dsDNA [155]. The HIN domain of IFI16 recognizes DNA ligands in a length-dependent manner [156]. Activated IFI16 induced the expression of IFNβ through IRF3 activation and the production of NF-κB-dependent proinflammatory genes during infection with several DNA viruses, including HIV, HSV-1, and VACV (Figure 2) [155,157,158,159]. IFNβ expression induced by transfection with VACV DNA was dependent upon STING, TBK1, and IRF3, but not TLRs, ZBP1, or Pol III. IFI16 physically interacted with STING, and BMDMs lacking STING failed to trigger IFNβ secretion in response to viral DNA [159]. IFI16 deficiency inhibited IRF3 activation- and NF-κB-dependent gene production induced by transfected DNA, and during infection with either MVA or HSV-1 [155,159]. During VACV infection, IFI16 shuttled from the nucleus of keratinocytes to viral factories in the cytosol for viral DNA recognition. Infection with the highly attenuated VACV strain MVA, but not with VACV-WR, induced IFI16-dependent CCL5 and ISG56 expression. These data indicate that VACV-WR can inhibit the IFI16 pathway, presumably via one or more of the genes missing in MVA [155]. The precise mechanisms of how VACV modulates IFI16-mediated DNA sensing are unclear, but viral immunomodulators that target downstream signaling of STING likely play a role.

### 4.4. DNA-Dependent Protein Kinase

DNA-dependent protein kinase (DNA-PK) is best studied for its involvement in DNA repair and V(D)J recombination [160]. However, in addition to this well-documented activity, DNA-PK can also recognize cytosolic DNA [161]. As a heterotrimeric complex, DNA-PK is comprised of the catalytic subunit DNA-PKcs and the Ku heterodimer, consisting of Ku70 and Ku80 subunits. Upon exposure to exogenous DNA, Ku70 translocated from the nucleus to the cytosol and triggered the production of type III IFN (IFNλ1), which involved STING and TBK1 as mediators. This activity was associated with the activation of the transcription factors IRF1, IRF3, and IRF7 (Figure 2) [161,162]. DNA-PK-dependent DNA sensing was shown to contribute to the initiation of the immune response to VACV in fibroblasts. Supporting this contribution, DNA-PK deficiency in either cell lines or mice significantly impaired the induction of IFNβ and IL-6 in response to infection with MVA or HSV-1 [163].

#### Poxvirus Evasion of DNA-PK

VACV expresses two related proteins, called C16 and C4 in the WR strain, that target the DNA-PK-mediated DNA sensing pathway (Figure 2). These proteins share about 54% amino acid identity in their C-terminal region [164,165,166]. The C-terminal region of C16 interacts directly with the Ku heterodimer of the DNA-PK complex to prevent DNA-PK binding to DNA, ultimately reducing the production of cytokines and chemokines. Consistent with this in vitro observation, mice infected with C16-deficient VACV, produced more cytokines and chemokines than mice infected with wild type VACV [164]. As with C16, VACV protein C4 also interacts with Ku and blocks DNA binding through its C-terminal domain, resulting in decreased IRF3 phosphorylation. Additionally, C4 inhibits the recruitment and activation of immune cells and suppresses cytokine production, such as IL-6, both in vitro and in vivo [165]. While C16 and C4 have overlapping functions, viruses with deletions of only one of these proteins were still attenuated in mouse infection models. The two most likely interpretations of these data are that there might be currently unrecognized non-redundant functions between the two proteins, or that C16 and C4 work in tandem by mass action [165].

### 4.5. DEAD Box Polypeptide 41

DEAD box polypeptide 41 (DDX41) was identified as a cytosolic sensor that recognizes diverse pathogen-derived nucleic acids, such as viral dsDNA, cyclic di-GMP, and cyclic di-AMP [167,168]. DDX41 is a member of the DEAD-box protein family and is composed of two RecA-like domains (DEADc and HELICc domains) and a zinc finger [169]. Upon binding dsDNA via its DEADc domain, DDX41 associates with STING and initiates the activation of NF-κB and IFN signaling pathways in myeloid dendritic cells (Figure 2) [167].

DDX41 directly recognized a repeating 70 bp motif (VACV 70mer), which is located in the inverted terminal repeat region of the VACV genome [159,167]. Knockdown of DDX41 or STING in THP-1 cells abolished the production of IFNβ and IL-6 in response to either the VACV 70mer or HSV-1 DNA [167]. It is unknown if poxviruses directly target DDX41, but it is possible that poxviral inhibitors that act downstream of STING activation can also interfere with this DDX41-mediated immune response (see Poxvirus Evasion of STING).

## 5. Toll-Like Receptor-Mediated Poxvirus Recognition and Poxvirus Antagonists

Toll-like receptors (TLRs) are a family of PRRs, which derive their name from their homology with the *Drosophila Toll* gene [170]. In *Drosophila*, activation of the Toll pathway by ligands from Gram-positive bacteria or fungi triggers cellular immunity and production of antimicrobial peptides [171,172,173]. Toll-like receptors function as PRRs to initiate signaling cascades important for host defense against many pathogens. There are 13 currently known TLRs in mammals (TLR1 to TLR13), although humans only possess TLRs 1 through 10 [174].

TLRs are type I integral membrane glycoproteins expressed in both immune cells and non-immune cells such as fibroblasts and endothelial cells. These receptors have a common architecture, with an N-terminal extracellular leucine-rich repeat-containing ectodomain, which is responsible for the recognition of PAMPs, a single transmembrane helix, and a C-terminal cytoplasmic Toll/interleukin-1 receptor (TIR) homology domain [175]. TLRs localize to the plasma membrane of the cell surface (TLRs 1, 2, 4, 5, 6, and 10) or to various intracellular compartments (TLRs 3, 7, 8, 9, 11, 12, and 13), such as the endoplasmic reticulum (ER), endosome, lysosome, and endolysosome [176]. This cellular localization is one determinant of the PAMPs sensed by TLRs [176]. Once activated, TLRs typically activate downstream effectors through either adaptor proteins, typically myeloid differentiation primary response gene 88 (MyD88) or TRIF.

**Figure 3:**
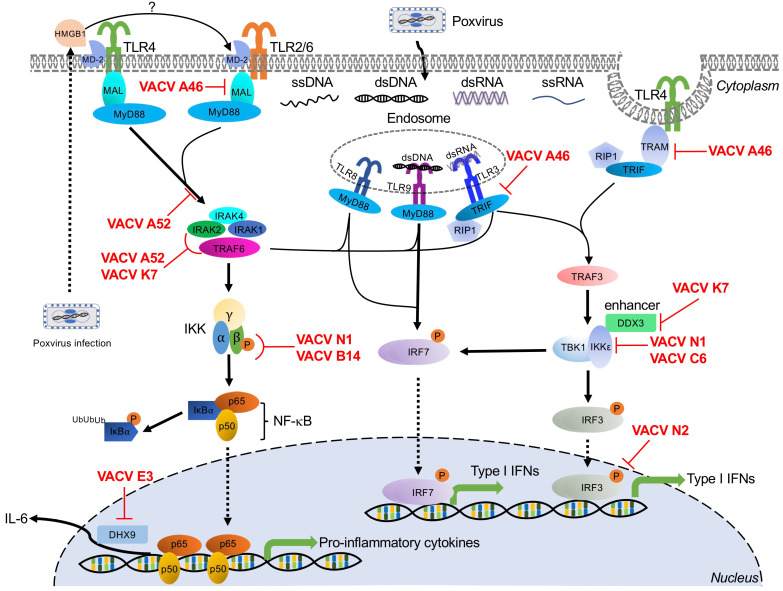
TLR family-mediated signaling pathways and poxvirus antagonists. TLR sensors involved in the recognition of poxviral infections are indicated in their subcellular localization. The signaling cascades induced by these TLRs are denoted by black arrows to indicate transduction or activation. Poxvirus-encoded viral antagonists and their targeted signaling molecules are shown in red. Abbreviations used in this figure include DDX3: Asp-Glu-Ala-Asp (DEAD) box polypeptide 3; DHX9: DExH-Box helicase 9; dsDNA: double-stranded DNA; dsRNA: double-stranded RNA; HMGB1: high mobility group box protein 1; IκBα: inhibitor κBα; IKKα: IκBα kinase α; IKKβ: IκBα kinase β; IKKε: IκBα kinase ε; IKKγ: IκBα kinase γ; IL-6: interleukin-6; IRAK1/2/4: interleukin-1 receptor-associated kinase 1/2/4; IRF3/7: interferon regulatory factor 3/7; Mal: myD88-adapter-like; MD-2: myeloid differentiation factor 2; MyD88: myeloid differentiation primary response gene 88; NF-κB: nuclear factor kappa B; p65/p50: NF-κB heterodimer p50/p65 subunit; RIP1: receptor-interacting protein 1; ssDNA: single-stranded DNA; ssRNA: single-stranded RNA; TBK1: TRAF family member-associated NF-κB activator (TANK)-binding kinase 1; TLR2/3/4/8/9: Toll-like receptor 2/3/4/8/9; TNFα: tumor necrosis factor-alpha; TRAF3/6: tumor necrosis factor receptor-associated factor 3/6; TRAM: TRIF-related adapter molecule; TRIF: Toll/interleukin-1 receptor domain-containing adapter-inducing interferon-β; VACV: vaccinia virus.

TLR2/6, TLR4, TLR8, and TLR9 recruit myeloid differentiation primary response gene 88 (MyD88) to transduce their signaling cascades [41]. Activation of MyD88-dependent signaling induces proinflammatory cytokines and chemokines (Figure 3). TLR8-MyD88 and TLR9-MyD88 signaling pathways are also engaged in IFN induction through IFN regulatory factor 7 (IRF7) activation in dendritic cell (DC) subsets, such as the plasmacytoid DCs (pDCs) [177]. MyD88 recruits and interacts with interleukin 1 receptor-associated kinase 4 (IRAK4) to form a structure known as the Myddosome along with two other IRAK family members, IRAK1 and IRAK2. This complex activates tumor necrosis factor receptor-associated factor 6 (TRAF6) [178]. TRAF6-induced activation of TGF-β activated kinase 1 (TAK1) subsequently phosphorylates the IKKβ subunit of the canonical IκB kinase (IKK) complex [179], resulting in ubiquitination and proteasomal degradation of IκBα and release of NF-κB [180], leading to the production of proinflammatory cytokines (Figure 3) [181]. Notably, Toll/interleukin 1 receptor (TIR) domain-containing adapter protein (TIRAP, also known as Mal) and TRIF-related adaptor molecule (TRAM) are further required for bridging MyD88 to TLR2/6 and TLR4.

The TRIF-dependent pathways are initiated through TLR3 and endosomal TLR4 and induce both inflammatory responses and type I IFNs through activation of TRAF6 or TRAF3, respectively [42]. TRIF associates with TRAF6 and RIP1 to activate the classical IKK complex through the activation of TAK1 kinase complex, resulting in the production of NF-κB-dependent proinflammatory cytokines and chemokines [181]. In contrast, TRIF interacts with TRAF3 to recruit the noncanonical IKK-related kinases TBK1 and IKKε for phosphorylation and activation of IRF3/IRF7, resulting in the subsequent induction of type I and type III IFNs (Figure 3) [182].

Several TLRs play roles in poxvirus infections, including endosomal TLR3, TLR8, and TLR9, and membrane-bound extracellular TLR2 and TLR4 (Figure 3).

### 5.1. TLR3

TLR3 localizes to the endosome and was the first characterized TLR to recognize nucleic acid [183]. The primary ligand of TLR3 dsRNA is recognized via the N-terminal ectodomain (ECD) [183,184,185]. After binding dsRNA, downstream signaling transduction is mediated through the TIR domain-containing adaptor-inducing interferon-β (TRIF) [186]. Activation of the TLR3-TRIF pathway leads to the production of NF-κB-dependent proinflammatory cytokines, and type I and type III IFNs (Figure 3) [183].

Paradoxically, when infected with VACV, TLR3^−/−^ mice showed decreased disease morbidity, accompanied by reduced VACV replication in the respiratory tract and impaired viral dissemination [25]. This TLR3 deficiency did not change the level of IFNβ but did reduce the levels of inflammatory cytokines, including IL-6, TNFα, and monocyte chemoattractant protein-1 (MCP-1/CCL2). These cytokine increases suggest that the increased morbidity and viral dissemination observed in TLR3-competent mice may be mediated through NF-κB-dependent inflammatory cytokines [25]. However, TLR3 has also been shown to improve post-exposure vaccine efficacy in response to ECTV infection in mice. BALB/c mice infected with ECTV can be cured by post-exposure vaccination with either the VACV-Lister or MVA strains up to three days after infection. However, post-exposure treatment with poly(I:C), an agonist of TLR and other dsRNA-binding proteins, either alone or in combination with traditional vaccination improved the efficacy of this treatment regimen by modulating TLR3 activation and IFNα induction [187]. Thus, the antiviral activity of TLR3 is complex and merits further investigation.

#### Poxvirus Evasion of TLR3

Poxviruses encode multiple Bcl-2-like proteins that target the TLR3 pathway, including A52, A46, N1, B14, K7, N2, and C6 proteins (Figure 3). Intriguingly, the first five of these proteins share homology, although as described below, they inhibit TLR3 through distinct mechanisms. A52 was shown to block TLR3-mediated activation of NF-κB induced by poly(I:C) stimulation through interaction with both interleukin 1 receptor-associated kinase 2 (IRAK2) and tumor necrosis factor receptor-associated factor 6 (TRAF6), disrupting the Mal-IRAK2 signaling complex or TRAF6-TAB1-containing complex, respectively [188]. In response to poly(I∶C), A46 was directly associated with TRIF, which acted as a TIR adapter for TLR3 signaling, in order to inhibit IRF3 activation and gene induction [189]. However, in the presence of poly(I:C), N1 physically interacted with components of the IKK complex and also associated with IKKε and TBK1, thereby inhibiting NF-κB activation and IRF3-mediated IFNβ responses [190]. B14, an immediate-early gene product of VACV, prevented phosphorylation of IKKβ, a component of the IKK complex, resulting in inhibition of NF-κB signaling induced by poly(I:C) [191,192]. K7 has been shown to interact with DEAD-box RNA helicase (DDX3) to inhibit TRIF-induced IRF3/7 activation and also prevented IFNβ promoter induction at the level of TBK1/IKKε [193]. VACV N2 localized to the nucleus and functionally inhibited IRF3 activity after translocation of IRF3 into the nucleus. Ultimately, this N2–IRF3 interaction decreased activation of the IFNβ promoter in response to poly(I:C) stimulation [152]. Finally, C6 has been reported to be a multifunctional interferon antagonist during VACV infection. In the context of the TLR3 pathway, C6 prevented TBK1- and IKKε-dependent IRF3 activation, resulting in inhibition of IFNβ promoter activation induced by poly(I:C) [151,152].

### 5.2. TLR8

TLR8 is broadly expressed in the endosomes of myeloid cells, such as monocytes, macrophages, and myeloid dendritic cells (DCs) [194,195]. TLR8 is non-functional until it undergoes proteolytic processing to generate a functional receptor in the endosome [195]. Activation of this Toll-like receptor is traditionally mediated through recognition of uridine- and guanosine-rich single-stranded RNA (ssRNA) of either bacterial or viral origin [196,197]. However, a recent report has shown that murine TLR8 can be activated by poxviral DNA or by synthetic poly(A) and poly(T) oligodeoxynucleotides both in vitro and in vivo. Either VACV infection or VACV DNA activated an NF-κB reporter expressed by HEK293 cells that expressed murine TLR8, but not in cells expressing murine TLR7. Similarly, siRNA-mediated knockdown of TLR8 reduced the secretion of IFNα in pDCs [198]. However, the underlying mechanism is yet to be determined.

Regardless of the ligand, binding transduces signaling through MyD88, ultimately resulting in the induction of IRF7 activation, IFN production, and NF-κB-dependent proinflammatory responses (Figure 3). In addition, VACV infection-induced IFNα production by pDCs in vitro and in vivo and TLR8-dependent pDC activation played an important role in the control of VACV infection in vivo [198].

#### Poxvirus Evasion of TLR8

VACV E3 has been reported to target TLR8-dependent pathways. In human monocytes, E3 reduced the expression of TLR8-dependent cytokines, including proinflammatory cytokines such as TNFα and IL-6, and chemokines such as interferon gamma-induced protein 10 (IP-10), and chemokine (C-C motif) ligand 5 (CCL5, also known as RANTES). In addition, E3 directly interacted with DExH-Box helicase 9 (DHX9) to antagonize IL-6 promoter activation [199].

### 5.3. TLR9

TLR9 was the first identified DNA sensor. It is localized in endosomes in a variety of cells including plasmacytoid dendritic cells (pDCs), B cells, neutrophils, monocytes, and some non-immune cells [200,201]. TLR9 undergoes proteolytic cleavage of its ectodomain to recognize unmethylated cytosine–guanosine (CpG)-rich DNA derived from bacteria or viral genomic dsDNA [200,202,203,204].

As the primary DNA-sensing TLR, TLR9 has been implicated in the response to multiple poxvirus infections. For example, TLR9 depletion dramatically decreased mouse survival after ECTV infection, which required TLR9 expression in DCs [26]. TLR9 depletion in these mice abolished ECTV-induced DC maturation and IFNα production, highlighting the important role of TLR9 when controlling ECTV infection [26]. In this ECTV model, the TLR9-MyD88-IRF7 pathway was essential for proinflammatory cytokine expression in CD11c^+^ cells and for the recruitment of inflammatory monocytes to the draining lymph node [142].

In response to MVA infection, TLR9-deficient or MyD88-deficient murine cDCs showed decreased production of IFNα and IFNβ compared to wild type cDCs [145]. TLR9 has also been implicated in the host response to fowlpox virus and MYXV through the above-described MyD88 signal transduction cascade [205,206].

#### Poxvirus Evasion of TLR9

To date, VACV E3 is the only described viral antagonist of the TLR9 pathway [206]. In cells treated with CpG-containing DNA, a TLR9 agonist, wild type VACV significantly inhibited IFNβ, TNF, and IL-12p70 (IL-12) production in murine pDCs. In contrast, infecting these cells with VACV lacking E3L reduced this inhibitory effect, which could be rescued by co-infection with wild type VACV. These results were also phenocopied if only the N-terminal Z-DNA binding (Zα) domain of E3 was deleted, demonstrating that this domain is important for TLR9 pathway inhibition [206].

### 5.4. TLR4

TLR4 was identified as a human Toll homolog in 1997 [207]. TLR4 predominantly recognizes bacterial lipopolysaccharide (LPS), mannuronic acid polymers, and teichuronic acid. TLR4 has also been shown to recognize viral glycoproteins, fusion proteins, and damage-associated molecular patterns (DAMPs) [208,209,210,211,212]. Whether TLR4 directly recognizes poxvirus ligands is not yet clear. One possibility is that TLR4 is indirectly activated after poxvirus infections. Previous work has demonstrated that infection with either VACV strains WR or MVA can lead to the extracellular release of high mobility group box protein 1 (HMGB1). Outside the cell, HMGB1 binds to myeloid differentiation factor 2 (MD-2), the extracellular adaptor of TLR4, to activate this pathway [213,214,215]. There is also some evidence that TLR4 may recognize a viral protein, potentially on the virion surface, rather than ligands released or generated during infection [216]. It is currently unclear if either or both of these mechanisms are the primary mode of TLR4 activation by poxviruses. After ligand binding, TLR4 dimerizes and initiates a signaling cascade via TLR adapter molecules MyD88 adaptor-like (Mal)/TIRAP, MyD88, TRAM, and TRIF, resulting in the production of inflammatory cytokines and type I IFNs (Figure 3) [217]. In response to VACV infection, TLR4 provided maximal protection against pulmonary VACV infection in a mouse model. Surprisingly, TLR4 dampened the cytokine response of bone marrow macrophages to VACV, and TLR4 deficiency promoted an increase in IFNβ and IL-6 production in the lungs [216].

#### Poxvirus Evasion of TLR4

VACV proteins A46, A52, K7, and N1 have been implicated in TLR4 evasion (Figure 3). A46 and A52 share amino acid sequence similarity with the Toll/interleukin-1 receptor (TIR) domain. The VACV A46 protein directly interacts with the TLR4, MyD88, Mal, TRIF, and TRIF-related adaptor molecule (TRAM), disrupting receptor–adaptor interactions and inhibiting downstream signaling [218,219,220,221,222]. A46-deficient VACV showed attenuated virulence in a murine intranasal infection model [189]. A52 targets TLR4 signaling, potently suppressing both IL-1- and TLR4-mediated NF-κB activation by mimicking the dominant negative effect of a truncated version of MyD88 [223]. VACV K7 blocks both IRAK2 and TRAF6 activation to inhibit the TLR4–NF-κB signaling axis [193]. Finally, N1 has been shown to target the TRAF6-TBK1-IKK complex, interacting with IKKα and IKKβ to block NF-κB responses [190].

### 5.5. TLR2

TLR2, first identified in 1998 [170], is expressed on the surface of immune cells, such as monocytes, macrophages, dendritic cells, and NK cells. On the cell surface, TLR2 forms heterodimers with either TLR1 or TLR6 to recognize a wide array of distinct ligands including lipopeptides and lipoteichoic acid from bacteria, and fungal polysaccharides [224,225,226,227]. TLR2 also recognizes multiple viral ligands, including envelope glycoproteins and core proteins from cytomegalovirus, HIV-1, and hepatitis C virus [228,229,230]. Upon ligand binding, TLR2 interacts with the adaptor protein MyD88, ultimately inducing NF-κB-dependent inflammatory responses (Figure 3). While TLR2 has been implicated in the innate response to VACV infection [202,203], defining the poxvirus ligands is still an ongoing area of research. As with TLR4, poxviruses may indirectly initiate TLR2 signaling through HMGB1 and MD-2. This mechanism was proposed for TLR2 activation by an MVA-vectored tuberculosis vaccine candidate (MVA85A), supported by the observation that treating mouse PBMCs with anti-HMGB1 antibodies reduced in vitro production of chemokine (C-X-C Motif) ligand 2 (CXCL2) [214]. However, the more basic question of whether TLR2 can be activated through this HMGB1/MD-2 axis remains controversial [231,232]. Both VACV and UV-inactivated VACV elicited similar immune responses through the TLR2-MyD88 pathway [24]. TLR2 activation by UV-inactivated viruses that should not induce cell death and thus HMGB1 release suggests that TLR2 activation may instead be mediated by direct binding of an as yet undescribed poxvirus ligand. TLR2 deficiency in conventional DCs (cDCs) and T cells reduced the secretion of IL-6 in response to VACV infection compared to infected wild type cells [24,233]. Additionally, TLR2-mediated signaling is important for NK cell activation after VACV infection and critical to control VACV infection in mice [234].

#### Poxvirus Evasion of TLR2

VACV encodes multiple antagonists of TLR2-mediated signaling, including A46, N1, and E3 (Figure 3). A46 and N1 belong to a family of B cell lymphoma 2 (Bcl-2)-like proteins and contain experimentally confirmed Bcl-2 folds [219,235,236]. A46 physically interacted with diverse TIR domain-containing adaptor proteins including MyD88, ultimately preventing TLR2-mediated activation of IRF7 and IFNβ responses [237]. VACV N1 inhibited the TLR2-mediated activation of NF-κB through a direct association with components of the IKK complex [190,236]. Finally, VACV E3 was described to act downstream of this pathway to interact with the DExD/H-box helicase DHX9 to inhibit DHX9-mediated enhancement of NF-κB-dependent IL-6 promoter activation [199].

## 6. Inflammasome Recognition of Poxviruses and Poxvirus Antagonists

**Figure 4:**
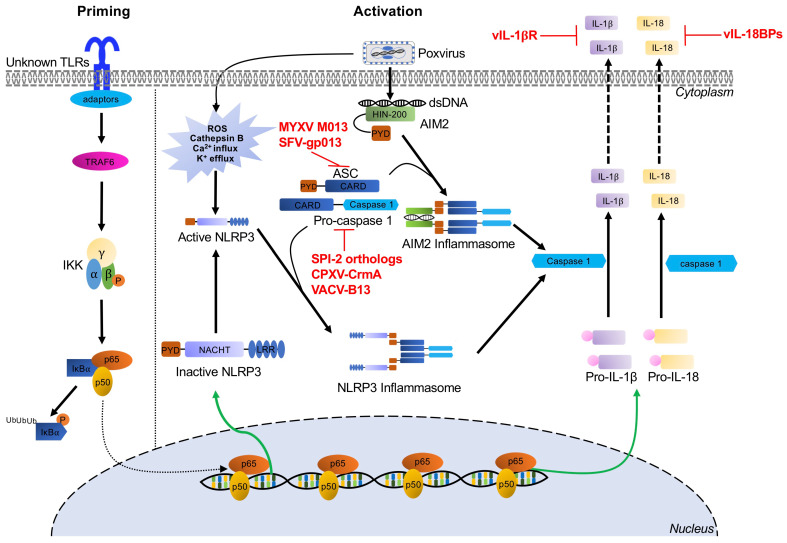
Inflammasome-mediated signaling pathways and poxvirus antagonists. NLRP3 and AIM2 inflammasome-mediated recognition of poxvirus infection and the priming and activation pathways for maturation and secretion of IL-1β and IL-18 effectors are indicated by black arrows. Poxviruses express several viral inhibitors or viral homologs of cellular proteins (shown in red) to interfere with these inflammasome pathways at different stages. Abbreviations used in this figure include AIM2: absent in melanoma 2; ASC: apoptosis-associated speck-like protein containing a CARD; CARD: caspase activating and recruiting domains; CPXV: cowpox virus; CrmA: cytokine response modifier A; dsDNA: double-stranded DNA; HIN-200: hematopoietic interferon-inducible nuclear proteins with a 200 amino acid repeat; IKKα: IκBα kinase α; IKKβ: IκBα kinase β; IKKγ: IκBα kinase γ; IL-18: interleukin-18; IL-1β: interleukin-1β; IκBα: inhibitor κBα; LRR: leucine-rich repeats; NF-κB: nuclear factor kappa B; NLRP3: NOD, LRR and pyrin domains-containing protein 3; NOD; nucleotide binding and oligomerization domain; p65/p50: NF-κB heterodimer p50/p65 subunit; PYD: pyrin domain; RFV: rabbit fibroma virus; ROS: reactive oxygen species; SPI-2: serine proteinase inhibitor 2; TLRs: Toll-like receptors; TRAF6: tumor necrosis factor receptor-associated factor 6; VACV: vaccinia virus.

Inflammasomes are multiprotein signaling complexes responsible for the production of proinflammatory cytokines and the induction of pyroptosis, an inflammatory lytic programmed cell death, to halt viral replication and induce nearby cells to adopt antiviral states [238]. Inflammasome activation mediates the conversion of inactive precursor proteins pro-interleukin (IL)-1β and pro-IL-18 into the bioactive forms IL-1β and IL-18, which play important roles in host defense against a variety of bacterial, fungal, and viral infections [239,240]. Certain PRRs have been implicated in canonical inflammasome assembly, including NOD-like receptors and AIM2-like receptors [241,242]. After PAMP and cell damage-associated signal recognition, adaptors are recruited, such as apoptosis-associated speck-like protein (ASC) [243]. This process results in cytokine secretion and pyroptosis as proteolytically active caspases mediate the maturation and secretion of proinflammatory cytokines IL-1β and IL-18, while cleavage of gasdermin-D (GSDMD), a key pyroptotic substrate of inflammatory caspases, induces pyroptosis (Figure 4) [244]. Specifically, the inflammasome proteins NACHT, LRR, and PYD domains-containing protein (NLRP3) and AIM2 have been implicated in the recognition of poxvirus infections [29,30,245].

### 6.1. The NLRP3 Inflammasome

NLRP3 acts as the intracellular sensor component of the NLRP3 inflammasome and detects a broad range of both PAMPs and host-derived activating signals (endogenous damage-associated molecular patterns, DAMPs). NLRP3 is expressed in innate immune cells and non-immune cells, including macrophages, neutrophils, and epithelial cells [246]. This sensor is comprised of an N-terminal pyrin domain (PYD), a central NACHT domain, and C-terminal leucine-rich repeat domains. NLRP3 inflammasomes must be primed before they are activated. This priming step can be mediated by a variety of signals including TLR or NLR ligands, which activate NF-κB. NF-κB then upregulates NLRP3 expression to levels sufficient to permit inflammasome assembly [247,248,249]. Once the PAMP or DAMP ligand is bound, oligomerized NLRP3 recruits ASC through homotypic PYD–PYD interactions and activates caspase 1, thereby triggering the secretion of the proinflammatory cytokines IL-1β and IL-18 (Figure 4). During MVA infection, crosstalk between the NLRP3 inflammasome and TLR2-TLR6-MyD88 has been documented to mediate the expression and processing of IL-1β in macrophages both in vivo and in vitro [29]. Furthermore, in a keratinocyte model of MVA infection, IL-1β secretion was reduced in the presence of pyrrolidine dithiocarbamate, BAPTA tetrakis (acetoxymethyl ester), and glibenclamide, suggesting that intracellular Ca^2+^ levels and K^+^ efflux may be involved in NLRP3 inflammasome activation [250].

#### Poxvirus Evasion of the NLRP3 Inflammasome

To inhibit NLRP3 activity, poxviruses target different stages of inflammasome assembly and processing, as well as inhibiting the secretion or the function of IL-1β and IL-18. Multiple poxviruses encode viral pyrin-only proteins (PYD/vPOP), which have homology with the ASC-PYD domain [18,251]. For example, one of these proteins, MYXV-M013, directly interacts with ASC-1 to inhibit NLRP3–ASC-1 interactions, thereby inhibiting activation of caspase-1 and the secretion of IL-1β and IL-18 [18,245,252,253]. Similarly, gp013 from rabbit fibroma virus (RFV) interacts with ASC to interfere with PYD-mediated activation of caspase-1 [251]. Poxviruses also encode serine proteinase inhibitor 2 (SPI-2) orthologs, including cytokine response modifier A (CrmA) from cowpox virus and VACV-B13. These proteins act as substrate mimics to inhibit caspase-1 activity, thus preventing proteolytic processing of IL-1β [254,255,256]. Additionally, poxviruses have evolved secreted viral IL-1β receptors (vIL-1βR), such as VACV-B15, CPXV-B14, and ECTV-191, which prevent IL-1β binding with the host receptors [257,258]. Similarly, viral IL-18 binding proteins (e.g., molluscum contagiosum virus -54L and VACV-C12) compete with the IL-18 cognate receptor for IL-18 binding [259,260,261].

### 6.2. The AIM2 Inflammasome

AIM2 belongs to the pyrin and HIN protein (PYHIN) family of proteins and is comprised of an N-terminal PYD domain, which recruits ASC through PYD–PYD interactions, and a C-terminal HIN200 domain, which is essential for recognition and binding cytosolic DNA [262,263,264,265]. Upon recognition of DNA, AIM2, ASC, and procaspase 1 form the AIM2 inflammasome. This inflammasome activates caspase 1 to initiate proinflammatory cytokine processing, resulting in maturation and secretion of IL-1β and IL-18 (Figure 4) [30,266,267].

The AIM2 inflammasome has been shown to recognize multiple viral infections including VACV, mouse cytomegalovirus, and influenza A virus [268,269,270,271]. For example, in cells derived from AIM2^−/−^ mice infected with VACV, IL-1β release and maturation, as well as caspase-1 cleavage, were all reduced [30,268]. Similarly, AIM2 knockdown in human primary keratinocytes almost completely abolished IL-1β and IL-18 production during MVA infection. In contrast, NLRP3 knockout only slightly reduced IL-1β secretion [250]. While these data clearly establish a role for the AIM2 inflammasome in response to poxvirus infection, no direct poxvirus inhibitors are currently known. However, the inhibitors of IL-1β and IL-18 activity, discussed in the previous section, are likely effective at preventing the activity of these downstream effectors of AIM2 inflammasome activation.

## 7. Conclusions and Outlook

In this review, we have discussed multiple PRRs and their roles in sensing poxviruses infections and subsequently initiating innate immune responses. Individual PRRs have unique molecular mechanisms for sensing ligands and triggering antiviral responses via diverse adapters and effectors. Furthermore, redundancy, cooperation, and crosstalk among the various PRRs increase this complexity. This crosstalk and redundancy in immune pathways are also reflected in the viral antagonists, with multiple PRR pathways targeted by the same viral proteins. For example, E3 sequesters the dsRNA and blocks the activation of PKR, OAS/RNase L, and TLRs [68,95,199,206]. However, it is currently an open question as to whether these multiple functions, for E3 and other antagonists, are truly redundant, or if there are situational differences in the activity of E3 against various pathways. Thus, the overall picture of host recognition of poxviruses is multifaceted and far from clear. Investigation of the cooperation and crosstalk between PRRs will help define the innate immune network(s) elicited by these various PRRs both individually and in combination.

On the other side of this battle, the specific poxviral ligands are not yet known for all of these PRRs. Identification of these ligands, characterization of their structures or motifs, and their interactions with the sensors themselves are necessary to reveal how the signal is initiated by receptors during infection of poxviruses. Furthermore, the molecular mechanisms underlying cell type-specific or virus-specific recognition and signaling by certain PAMPs during poxvirus infections are still largely unknown. Finally, it is becoming more apparent that the diversity in the poxvirus family is also reflected by the range of activities discovered for individual viral gene orthologs, and their implications for viral host range and virulence. This observation is most strongly supported for rapidly evolving genes such as viral immune regulators and their implications for virus host range and virulence [4,72,78,272]. Therefore, the data presented in this view representing the response to a handful of poxviruses may not capture the full spectrum of poxvirus responses, and viral orthologs from other poxviruses should also be examined.

## Abbreviations

ADAR1: adenosine deaminase acting on RNA 1; ADP: adenosine diphosphate; AIM2: absent in melanoma 2; AMP: adenosine monophosphate; AP1: activator protein 1; ASC: apoptosis-associated speck-like protein containing a CARD; ATP: adenosine triphosphate; Bcl-2: B cell CLL/lymphoma 2; CARD: caspase activation and recruitment domains; CCL5: chemokine (C-C motif) ligand 5; cGAS: cyclic GMP-AMP synthase; CNPV: canarypox virus; COP: Copenhagen; CPXV: cowpox virus; CPXV: cowpox viruses; CREP: constitutive repressor of eIF2α phosphorylation; CrmA: cytokine response modifier A; CTD: carboxy-terminal domain; CXCL2: chemokine (C-X-C Motif) ligand 2; DAMPs: damage-associated molecular patterns; DDX3: Asp-Glu-Ala-Asp (DEAD) box polypeptide 3; DDX41: Asp-Glu-Ala-Asp (DEAD) box polypeptide 41; DHX9: DExH-Box helicase 9; DNA-PK: DNA-dependent protein kinase; DNA-PKcs: DNA-dependent protein kinase catalytic subunit; dsDNA: double-stranded DNA; dsRBD: dsRNA binding domain; dsRNA: double-stranded RNA; ECTV: ectromelia virus; eIF2: eukaryotic translation initiation factor 2; eIF2B: eukaryotic translation initiation factor 2B; eIF2α: alpha-subunit of the eukaryotic translation initiation factor 2; ER: endoplasmic reticulum; GADD34: growth arrest and DNA damage-inducible protein 34; GDP: guanosine diphosphate; GMP: guanosine monophosphate; GTP: guanosine-5′-triphosphate; HSV-1: herpes simplex virus 1 HIN-200: hematopoietic interferon-inducible nuclear proteins with a 200 amino acid repeat; HMGB1: high mobility group box protein 1; IFI16: interferon-γ inducible protein 16; IFNAR: type I interferon receptor; IFNs: interferons; IKKα: IκBα kinase α; IKKβ: IκBα kinase β; IKKε: IκBα kinase ε; IKKγ: IκBα kinase γ; IL-18: interleukin-18; IL-1β: interleukin-1β; IL-6: interleukin-6; IRAK1/2/4: interleukin-1 receptor-associated kinase 1/2/4; IRF1/3/7: interferon regulatory factor 1/3/7; ISGs: IFN-stimulated genes; IκBα: inhibitor κBα; KD: kinase domain; LPS: lipopolysaccharide; LRRFIP1: leucine-rich repeat flightless-interacting protein 1; Mal: myD88-adapter-like; Malp2: macrophage-activating lipopeptide 2; MAVS: mitochondrial antiviral-signaling protein; MCP1: monocyte chemoattractant protein 1; MD-2: myeloid differentiation factor 2; MDA5: melanoma differentiation-associated protein 5; MIP2: macrophage inflammatory protein 2; MLKL: mixed lineage kinase-like; MRE11: meiotic recombination 11; mTORC1/2: mammalian target of rapamycin complex 1/2; MVA: modified vaccinia virus Ankara; MyD88: myeloid differentiation primary response gene 88; MYXV: myxoma virus; NF-κB: nuclear factor kappa B; NLRP3: NOD-, LRR- and pyrin domain-containing protein 3; NOD; nucleotide binding and oligomerization domain; OAS: 2′-5′-oligoadenylate synthetases; OASL: OAS like; p65/p50: NF-κB heterodimer p50/p65 subunit; PAMPs: pathogen-associated molecular patterns; PKR: protein kinase R; PRRs: pattern recognition receptors; PYD: pyrin domain; LRR: leucine-rich repeats; PYHIN: pyrin and HIN domain; RFV: rabbit fibroma virus; RHIM: RIP homotypic interaction motif; RIG-I: retinoic acid-inducible gene I; RIP1: receptor-interacting protein 1; RIPK: receptor interacting protein kinase; RLRs: RIG-I-like receptors; RNA pol III: DNA-dependent RNA polymerase III; RNase L: ribonuclease L; ROS: reactive oxygen species; SPI-2: serine proteinase inhibitor 2; ssDNA: single-stranded DNA; ssRNA: single-stranded RNA; STING: stimulator of interferon genes; TAK1: TGF-β activated kinase 1; TBK1: TRAF family member-associated NF-κB activator (TANK)-binding kinase 1; TIR: Toll/Interleukin-1 receptor; TIRAP: Toll/interleukin 1 receptor (TIR) domain-containing adapter protein; TLRs: Toll-like receptors; TNFα: tumor necrosis factor-alpha; TRAF3/6: tumor necrosis factor receptor-associated factor 3/6; TRIF: Toll/interleukin-1 receptor domain-containing adapter-inducing interferon-β; TRAM: TRIF-related adapter molecule; VACV: vaccinia virus; VARV: variola virus; vIL-18BPs: viral IL-18 binding proteins; vIL-1βR: viral IL-1β receptor; VIPER: viral inhibitory peptide of TLR4; vPOP: viral pyrin-only proteins; vSlfn: viral Schlafen; WR: Western Reserve; ZBP1: Z-DNA binding protein 1; Zα: Z-nucleic acid binding domain; 2-5As: 2′-5′-oligoadenylates; 2′3′ cGAMP: 2′3′ cyclic guanosine monophosphate–adenosine monophosphate; 5′ppp-dsRNA: 5′ triphosphate double-stranded RNA.
